# Normobaric hypoxia overnight impairs cognitive reaction time

**DOI:** 10.1186/s12868-017-0362-3

**Published:** 2017-05-15

**Authors:** Stephan Pramsohler, Stefan Wimmer, Martin Kopp, Hannes Gatterer, Martin Faulhaber, Martin Burtscher, Nikolaus Cristoph Netzer

**Affiliations:** 1 Hermann Buhl Institute for Hypoxia and Sleep Medicine Research, Ghersburgstr. 9, 83043 Bad Aibling, Germany; 2 Department of Sport Science, University Innsbruck, Fürstenweg 185, 6020 Innsbruck, Austria; 3 Division of Sports Medicine and Rehabilitation, Department of Medicine, University Ulm, Fürstenweg 185, Ulm, Germany

**Keywords:** Reaction time, Hypoxia, Cognition, Sleep, Extreme altitude

## Abstract

**Background:**

Impaired reaction time in patients suffering from hypoxia during sleep, caused by sleep breathing disorders, is a well-described phenomenon. High altitude sleep is known to induce periodic breathing with central apneas and oxygen desaturations, even in perfectly healthy subjects. However, deficits in reaction time in mountaineers or workers after just some nights of hypoxia exposure are not sufficiently explored. Therefore, we aimed to investigate the impact of sleep in a normobaric hypoxic environment on reaction time divided by its cognitive and motoric components. Eleven healthy non acclimatized students (5f, 6m, 21 ± 2.1 years) slept one night at a simulated altitude of 3500 m in a normobaric hypoxic room, followed by a night with polysomnography at simulated 5500 m. Preexisting sleep disorders were excluded via BERLIN questionnaire. All subjects performed a choice reaction test (SCHUHFRIED RT, S3) at 450 m and directly after the nights at simulated 3500 and 5500 m.

**Results:**

We found a significant increase of cognitive reaction time with higher altitude (p = 0.026). No changes were detected in movement time (p = n.s.). Reaction time, the combined parameter of cognitive- and motoric reaction time, didn’t change either (p = n.s.). Lower SpO_2_ surprisingly correlated significantly with shorter cognitive reaction time (r = 0.78, p = 0.004). Sleep stage distribution and arousals at 5500 m didn’t correlate with reaction time, cognitive reaction time or movement time.

**Conclusion:**

Sleep in hypoxia does not seem to affect reaction time to simple tasks. The component of cognitive reaction time is increasingly delayed whereas motoric reaction time seems not to be affected. Low SpO_2_ and arousals are not related to increased cognitive reaction time therefore the causality remains unclear. The fact of increased cognitive reaction time after sleep in hypoxia, considering high altitude workers and mountaineering operations with overnight stays, should be further investigated.

## Background

With the popularity of mountaineering even ill-prepared climbers tend to spend their nights during ascent at elevated altitudes [1, 2]. Climbing projects get more audacious, driven by excessive ambition and overestimation [3]. Also working operations at high altitude are still rising. This includes work at constructions or in mines, touristic enterprises and research work in observatories [4]. This requests a more detailed look at the consequences of high altitude sleep and successive performance of tasks.

It is well known that sleep quality decays increasingly with severe hypoxia. Especially periodic breathing and central apneas seem to disturb restorative sleep [5–10]. While ascending, alpinists and altitude workers experience their lowest oxygen levels during the night when arbitrary breathing does not happen. Therefore, symptoms of acute mountain sickness are likely to appear immediately after sleeping at a new altitude [11]. Several studies show that exposure to hypoxic environments affects reaction time [12–18]. Prolonged reaction to critical tasks at altitude could enhance the risk of injury. Reaction time is formed by a cognitive and a motoric part where the cognitive or “premotor” part seems to play a significant role [19]. It seems that from data provided about trauma and death during mountaineering, deficits in cognition form a considerable part. Between 1926 and 2006 1.3% of the attempts to reach the summit of Mount Everest ended fatal. From 113 deaths during this time, trauma is the most reported cause. Narrations from their previous health status suggest that the most common symptoms leading to this were profound fatigue and cognitive impairment [20].

If sleep in hypoxic condition causes a momentary impairment in reacting to simple tasks, this could be a cause for slow or wrong actions. More knowledge might be important not only for preventing accidents in mountaineers, but also in the above mentioned workers at high altitude.

The aim of this study is to investigate reaction time divided by its cognitive and motoric aspect in a standardized environment after sleep in high and extreme altitude. By using normobaric hypoxia, standardized conditions without environmental and constitutional influences are ensured.

## Methods

### Subjects

Five female and six male healthy students (mean age 21.0 ± 2.1 years) participated in the study. All Subjects have been recruited from the sports faculty of the University of Innsbruck and were very sportive. Subjects reporting any medical disorder, including sleep apnea (Berlin Questionnaire), smoking and pre-acclimatization to high altitude were excluded from participation.

### Protocol

According to Dykiert et al. [21], simple reaction time starts to be significantly impaired in altitudes over 4000 m. A normobaric room using an oxygen expulsion System (Low Oxygen Systems; Berlin-Buch, Germany) was used for the simulation. This allows reducing oxygen in the whole chamber to a minimum of 9.3% while keeping CO_2_ levels correspondingly low. As stated by Saugy et al. normobaric hypoxia demands a higher simulation to provoke similar effects as in hypobaric environments [22]. Since only a single exposure night in normobaric hypoxia was used, it seemed necessary to simulate 5500 m to provoke adequate reactions. In order to provide enough safety for the subjects and to be able to monitor a longer sleeping phase on 5500 m, a night at simulated 3500 m seemed necessary for pre-acclimatization.

**Fig. 1:**
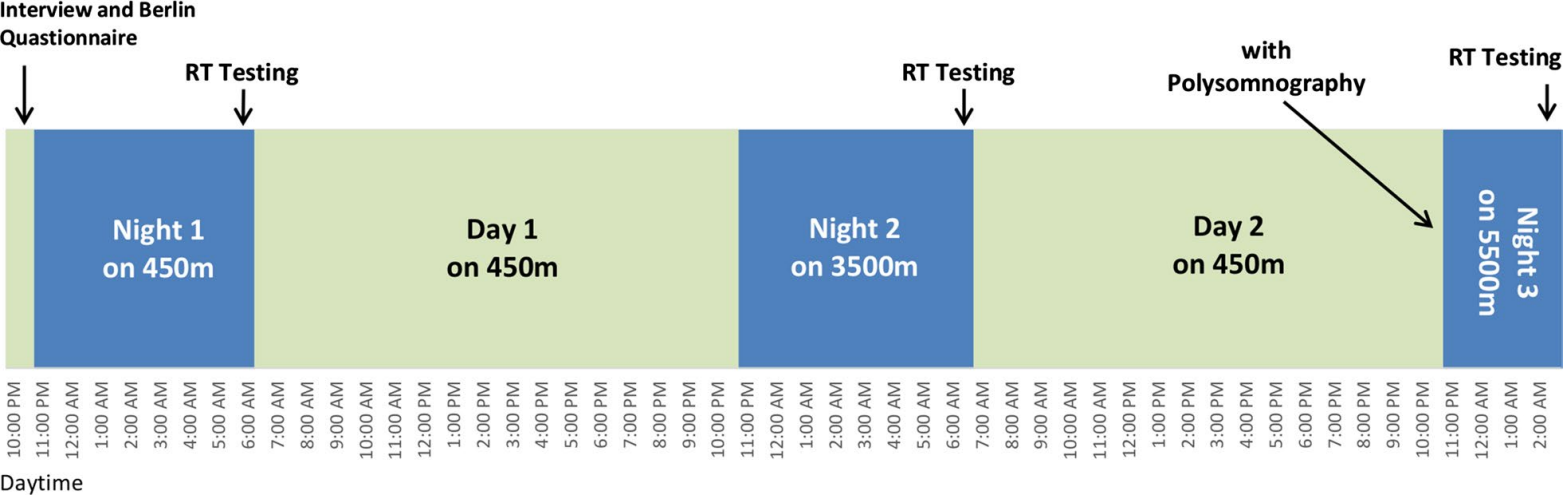
Study protocol. Study design with markings at every testing point. Wake and sleep phases correspond to the actual circadian periods of measurement (mean individual sleeping time at Night 1 and Night 2 = 7.50 h, Night 3 = 3.23 h) reaction testing is displayed as RT testing

Eleven subjects were asked to sleep for three successive nights in an altitude chamber. After every night a reaction testing (RT Schuhfried) was performed to assess motoric and cognitive reaction time. The testing took place directly after awakening, still inside the hypoxic room. Three different heights were chosen to determine changes in cognitive and motoric reaction time after sleep. The baseline testing took place at 450 m above sea level, corresponding to an inspiratory oxygen fraction (FiO_2_) of 20.93% (altitude at Bad Aibling, Germany). The second testing took place at a simulated altitude of 3500 m, equivalent to an FiO_2_ of 14,29% followed by night three at a simulated altitude of 5500 m (FiO_2_ = 9905%). All testing’s were conducted with respectively two subjects at the time. The study protocol is displayed in Fig. 1.

Every testing night began at 11:00 p.m. with the entering of the altitude room. Night one and night two were interrupted at 6:30 by the investigator, to perform the reaction testing, marking the end of hypoxic exposure. All subjects were still sleeping by then. Night three was monitored by a full 12 channel polysomnography. Polysomnoghraphy was mainly chosen as a monitoring tool for safety reasons. This way it was possible to react immediately if needed. Additionally, it allows looking for influencing factors regarding altitude sleep and changes in cognitive and motoric reaction. In night three polysomnoghraphic data was used to determine actual sleeping time instead of purporting a fix sleeping interval. The subjects were instructed to be aware of the symptoms of high altitude sickness. The Lake Louise Score was assessed in a standardized manor, directly after completing the testing sequence. In case of unbearable mountain sickness symptoms, subjects were free to disrupt the night sleep at any time. If sleep was interrupted, the choice reaction test was still completed directly after awakening and still in hypoxic conditions.

The protocol was designed to exclude the influence of constitutional differences after an exhaustive hike. The aim was to exclude environmental influences and therefore isolate the effect of sleep. Normobaric hypoxia was chosen to provide more safety for the personnel and the subjects. This way, they could leave the hypoxic rooms at any time without passing through a lock.

### Measuring

The reaction test (RT Schuhfried) measures the response time after both, a visual stimulus and a sound signal (Test form S3). The subjects were instructed to react as quickly as possible to the shown stimulus. The test score evaluates reaction time divided by its cognitive and motoric part needed to the respond to the shown stimulus (React when a yellow and a black dot are displayed while a sound is played simultaneously). Subjects were asked to press and hold the “SPACE” button on the keyboard during the whole testing time. When a relevant signal was displayed they were instructed to move to the “ENTER” key and back to the “SPACE” key as fast as possible. The elapsed time from the showing of the stimulus until leaving the “SPACE” button was measured as cognitive reaction time. The time needed for the movement from the “SPACE” key to pressing the “ENTER” key was measured as motoric reaction time. Obviously, the reaction time is the sum of motoric- and cognitive reaction time. The testing time was 10 min. Each subject was instructed by two learning phases each lasting 2 min. The twelve channel polysomnography on simulated 5500 m was carried out according to the American Academy of Sleep Medicine standard of 2007. Acute mountain sickness symptoms were only assessed orally.

### Data analysis

Data are presented as medians or means (were applicable) ± standard deviation (SD). Data analyses were performed with the SPSS statistical software package (PASW Statistics for Windows version 21.0, SPSS Inc., Chicago, IL, USA). An ANOVA for repeated measurements was applied to identify differences between the different heights. The Pearson test was used to determine correlations. Significance level was set at p < 0.05. A post hoc power analysis was performed with GPower 3.1.

## Results

**Fig. 2:**
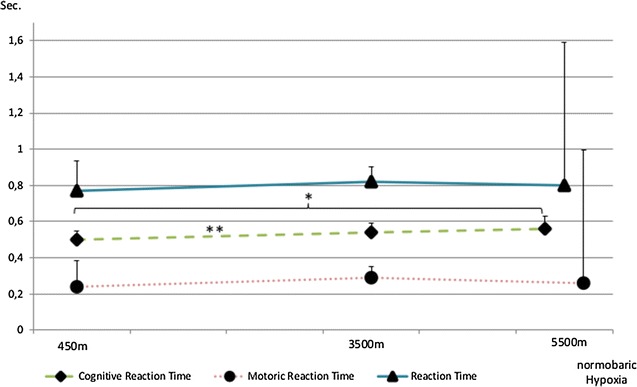
Changes in reaction time and its components, cognitive- and motoric reaction time over the three testing altitudes. Cognitive reaction time is prolonged with increasing altitude. Significant changes have been marked as follows (*p < 0.05; **p < 0.025). Data is displayed as medians with standard deviation; reaction time and its components are displayed in seconds (s) and altitude in simulated meters a.s.l (m)

We have seen a significant increase of cognitive reaction time with an increase in altitude as shown in Fig. 2 (p = 0.026). At 450 m median cognitive reaction time was 0.5 s ± 0.047 and increased from 0.54 s ± 0.05 at 3500 m up to 0.56 s ± 0.07 at 5500 m. The main statistical effects were detected between 450 and 3500 m as well as from 450 to 5500 m. No changes were detected in motoric reaction time (450 m = 0.24 s ± 0.14, 3500 m = 0.29 s ± 0.06 and 4500 m = 0.26 s ± 0.74, p = n.s.). The superordinate parameter of reaction time, didn’t change as well and was measured as 0.77 s ± 0.17 at 450 m, 0.82 s ± 0.08 at 3500 m and 0.8 s ± 0.79 at 5500 m (p = n.s.).

All subjects reported sleeping problems at 3500 m and quite severe periodic breathing episodes were measured on 5500 m simulated altitude with great individual variance. Male subjects reacted more distinct, i.e. had more apneas with increasing hypoxia than females (Mean Apnea/hypopnea index AHI, respiratory events/h: male = 121.65 ± 62.84, female = 59.59 ± 68.13, p = n.s.). No statistically significant gender differences could be detected.

**Fig. 3:**
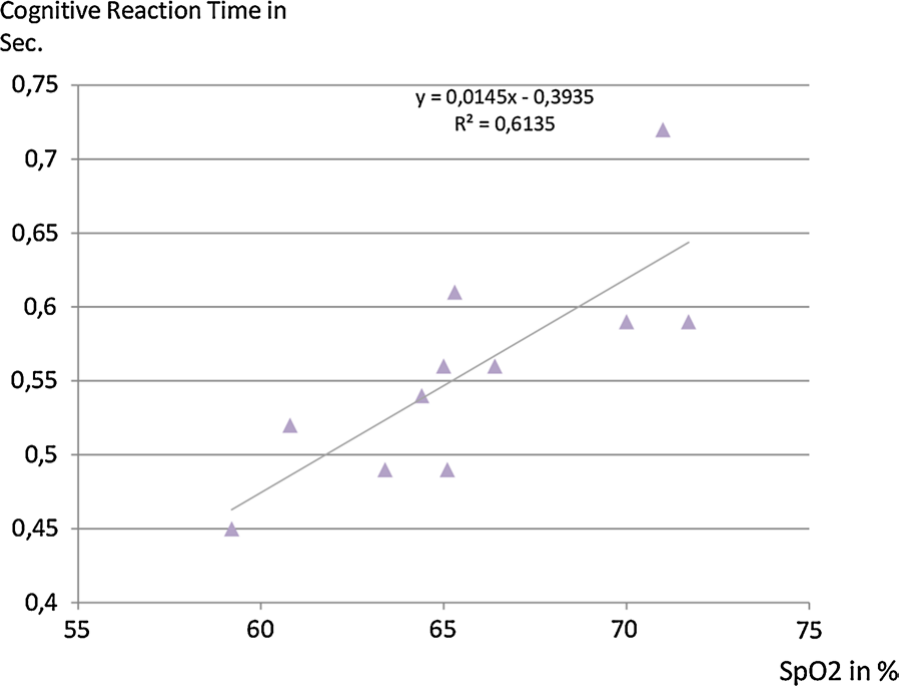
Correlation between cognitive reaction time and mean SpO_2_ overnight at 5500 m. High mean SpO_2_ overnight correlates with slow cognitive reaction time (R = 0.78, p = 0.004)

At an altitude of 5500 m individual mean peripheral oxygen saturation (SpO_2_) readings showed a positive correlation towards shorter cognitive reaction time (r = 0.78, p = 0.004). Low individual mean oxygen saturation was associated with a faster cognitive reaction (Fig. 3). Polysomnographic parameters show a severely impaired sleeping behavior at simulated 5500 m (Table 1). The parameter of AHI correlates with total sleeping time (r = 0.57, p = 0.003) as well as sleep phase 3 (r = 0.45, p = 0.022) and rapid eye movement sleep (REM) periods (r = 0.54, p = 0.004). Individuals who sleep longer have a significantly higher AHI, especially if they have more phase 3 and REM sleep (Table 1). A tendency regarding SpO_2_ and total sleeping time could be detected as higher SpO_2_ allows longer sleep or vice versa (r = 0.55, p = 0.079). The post hoc power analysis for the parameter cognitive reaction time accounted for an effect size of 0.97.

**Table 1:** Polysomnographic data at simulated 5500 m

ID	C-01	C-02	C-03	C-04	C-05	C-06	C-07	C-08	C-09	C-10	C-11
Sex (f/m)	m	f	f	m	f	m	f	m	f	m	m
TST (min)	155	108	40	103.5	347	373.5	122	334.5	135.5	157.3	260.9
N 1 (%)	3.9	9.3	33.8	11	13.1	7.5	12.3	24.8	10.5	15.9	27.9
N 2 (%)	44.5	39.8	47.5	63.3	52.9	66.7	75	52.8	39.9	54.5	48.5
N 3 (%)	37.7	50.9	18.5	18.8	20.3	60.5	12.7	22.4	41.7	21.6	19.8
REM (%)	13.9	0	0	6.8	13.7	9.6	0	0	7.7	7.9	3.7
CA (n)	382	53	111	312	291	907	4	759	0	163	87
CH (n)	15	7	1	17	189	40	12	39	13	9	20
AHI (n/h)	153.68	33.33	168	190.72	83	152.13	7.87	143.139	5.76	65.61	24.61
HF (bpm)	58	92.9	82.2	89.6	71.9	62.8	73.8	91.6	73.8	79.8	72.48
SpO_2_ (%)	66.4	60.8	65.1	59.2	71.7	70	64.4	65.3	65	71	63.4

*TST* total sleeping time, *N1* non REM 1, *N2* non REM 2, *N3* non REM 3, *REM* rapid eye movement sleep, *CA* central apneas, *CH* central hypopneas, *AHI* apnea hypopnea index, *HF* heart frequency, *SpO*_*2*_ peripheral oxygen saturation

## Discussion

To our knowledge, this is the first study on reaction time and its single components, cognitive reaction time and motoric reaction time, after sleep in normobaric hypoxia assessed in a standardized environment. Until now, most studies examined this matter in real altitude with great environmental and subjects-related, constitutional influences [12, 13]. The greater parameter of reaction time was not affected as much by low FiO_2_ levels during the night. Nevertheless, data show that high altitude sleep has a significant impact on cognitive reaction time.

In the present sample, cognitive reaction time decreased constantly throughout the trial with decreasing FiO_2_ levels. This is in line with several authors postulating the impact of hypoxemia on cognitive functioning when sleeping in hypobaric hypoxia [12–18]. A strong promoter for cognitive dysfunction after acute hypoxia exposure of lowlanders could be the raised inflammation markers as C-Reactive Protein and Interleukin as suggested by Sheng and Goldstein [13, 23]. In contradiction to other studies, in our sample poor cognitive reaction was not associated with low peripheral SpO_2_ levels during the night at 5500 m simulated altitude. This leads us to believe that low saturation levels are not the only factor to affect cognitive reaction. Subjects with shorter total sleeping time showed a tendency towards lower SpO_2_ levels and during this short exposure time, less impaired sleep. Short exposure in combination with a quite light sleep may not impair cognitive functioning as much as longer periods of deeper sleep. This observation requires more research and is not sufficiently probed by our data.

Motoric reaction shows a high individual variability and changes did not reach statistical significance. As anaerobic energy systems in the skeletal muscle outbalance aerobic mechanisms in such a short reaction testing and the movement intensity is quite low, it is not surprising that motoric reaction is not prolonged significantly by low saturation levels [24].

As expected, sleep quality was severely impaired in the present study group at simulated 5500 m altitude. A piled occurrence of periodic breathing and a quite short mean total sleeping time of 3 h and 14 min were measured at 5500 m. Progressively, impaired sleep quality with increasing altitude concurs with previous findings of various study groups [8, 10, 25]. Lower medium oxygen saturation during the night was associated with a better cognitive performance, which contradicts our expectations. Sleep related data did not show conclusive relations between saturation levels, AHI, sleeping phases and total sleeping time. Nevertheless, data leads us to believe that periodic breathing (i.e. high AHI) may have a SpO_2_ stabilizing function as hypothesized by Küpper et al. [7]. Subjects suffering from severe periodic breathing showed a higher SpO_2_, a longer and deeper sleep with more periodic episodes, which supposedly provoked more severe impairment in cognitive reaction time.

The screening for gender related differences in reaction time didn’t show any correlations. Non-significant differences were seen regarding AHI and total sleeping time. It is known that female hormone during menstrual cycle is an important factor in the regulation of stress-related behaviors and inflammatory signaling pathways [26, 27]. The lack of significant differences is supposedly owed to the small sample size.

Of course some restrictions influence our study outcome. As expected, many of the subjects only slept for a few hours at a simulated altitude of 5500 m. This way, only a short period of altitude sleep remained to be analyzed by the investigators. Furthermore, due to the very bumpy sleeping behavior at simulated 5500 m, difficulties in the evaluations of the sleeping related parameters have to be recognized. Sometimes it was difficult to divide body movement-waves and ECG-waves to identify sleeping stages. This fact could account for the inconclusive sleep data. A first effect of acclimation and ventilatory adaption during the first night could have affected the performance in the second testing. Nevertheless, this acclimatization night was necessary for safety reasons. For a complete assessment of sleep and the influence at reaction testing, 12 channel polysomnography would have been needed at night one and two as well. Since the study was conducted in normobaric environment there are less disruptive factors. Nevertheless, the study protocol allows only a restricted transfer to real altitude conditions.

## Conclusion

Cognitive reaction time is impaired after sleeping in high altitude in normobaric conditions. The relations between sleep quality, low SpO_2_ and cognitive functioning still remain unclear and need further investigation. Cognitive impairment could be among one of the first effects caused by hypoxia as already hypothesized by Roach and colleagues and it could be one of the most fatal [28].
